# Impacts and Potential Mitigation of Road Mortality for Hedgehogs in Europe

**DOI:** 10.3390/ani10091523

**Published:** 2020-08-28

**Authors:** Lauren J. Moore, Silviu O. Petrovan, Philip J. Baker, Adam J. Bates, Helen L. Hicks, Sarah E. Perkins, Richard W. Yarnell

**Affiliations:** 1 School of Animal, Rural and Environmental Sciences, Nottingham Trent University, Southwell NG25 0QF, Nottinghamshire, UK; adam.bates@ntu.ac.uk (A.J.B.); helen.hicks@ntu.ac.uk (H.L.H.); richard.yarnell@ntu.ac.uk (R.W.Y.); 2 Department of Zoology, University of Cambridge, The David Attenborough Building, Cambridge CB2 3QZ, Cambridgeshire, UK; sop21@cam.ac.uk; 3 School of Biological Sciences, University of Reading, Reading RG6 6AH, Berkshire, UK; p.j.baker@reading.ac.uk; 4 School of Biosciences, Cardiff University, Sir Martin Evans Building, Cardiff CF10 3AX, UK; perkinss@cardiff.ac.uk

**Keywords:** road mortality, collision, fragmentation, movement, demography, population viability, mitigation, road ecology, hedgehogs

## Abstract

**Simple Summary:**

The environmental impacts of transport infrastructure are attracting substantial research focus and road-induced mortality of wildlife is perhaps the most conspicuous impact of roads. Hedgehogs are a common victim of traffic collisions in Europe and several hedgehog species are showing marked population declines across their range. This review aims to consolidate current knowledge on the impacts of road mortality on the viability of populations of the five hedgehog species in Europe and identify research gaps. Previous studies have shown that roads are a major source of mortality for hedgehogs and that individuals with greater net movement, generally males, have the greatest likelihood of mortality. Road mortality also contributes to population isolation. More research is needed into how different individuals perceive, use and cross roads, as well as the efficacy of different mitigation measures (e.g., wildlife crossing structures) designed to reduce road mortality and population isolation. Assessing whether local hedgehog populations are at risk of extirpation or further declines due to road mortality is a prerequisite for effective conservation in environments affected by continuously developing road networks.

**Abstract:**

Transport infrastructure is a pervasive element in modern landscapes and continues to expand to meet the demands of a growing human population and its associated resource consumption. Road-induced mortality is often thought to be a major contributor to the marked declines of European hedgehog populations. This review synthesizes available evidence on the population-level impacts of road mortality and the threat to population viability for the five hedgehog species in Europe. Local and national studies suggest that road mortality can cause significant depletions in population sizes, predominantly removing adult males. Traffic collisions are a probable cause of fragmentation effects, subsequently undermining ecological processes such as dispersal, as well as the genetic variance and fitness of isolated populations. Further studies are necessary to improve population estimates and explicitly examine the consequences of sex- and age-specific mortality rates. Hedgehogs have been reported to use crossing structures, such as road tunnels, yet evaluations of mitigation measures for population survival probability are largely absent. This highlights the need for robust studies that consider population dynamics and genetics in response to mitigation. In light of ongoing declines of hedgehog populations, it is paramount that applied research is prioritised and integrated into a holistic spatial planning process.

## 1. Introduction

The last century has been characterised by intense modification of the natural landscape, and road networks are now pervasive in most landscapes on Earth [1,2]. Interest in the ecological impacts of roads has grown since the mid-20th century, with formal recognition of a new field, road ecology, by Forman and Alexander in 1998 [3]. This branch of ecological research has revealed the extensive role that roads play in direct and indirect habitat loss and alteration. Traffic noise, light pollution and chemical pollution (salt, heavy metals, herbicides) are all identified as important correlates of habitat modification, fragmentation and changes in animal movement in road-dominated environments [4].

Perhaps the most conspicuous impact of roads are wildlife–vehicle collisions (WVCs) that result in the death of billions of animals worldwide every year [5]. Biological characteristics of the animals themselves (e.g., age, sex and movement), biotic factors (e.g., time of day, season), traffic and road characteristics (e.g., traffic volume, road width, tortuosity) and environmental characteristics (e.g., topography, neighbouring habitat structure) all interact to form a species-specific spatiotemporal distribution of WVCs [6]. The consequences of road mortality are typically two-fold: (1) direct depletion of individuals from a population and/or (2) fragmentation of populations and reduced gene flow [7,8,9]. Importantly, these consequences can alter meta-population structure and population fitness, in turn increasing the risk of local extinction [10]. Roads are therefore considered responsible for the nationwide decline of certain species and a limiting factor in the recovery of others [11,12]. The growing literature on road ecology has been largely motivated by WVCs that are of legislative or conservation concern and/or which give rise to economic or human safety issues, such as collisions with deer [6]. In comparison, fewer studies have examined smaller mammal species, such as hedgehogs.

There are five species of hedgehog with all or part of their range in Europe, although hedgehog taxonomy has been debated due to contradictions between molecular and morphological phylogenies [13]. The West European hedgehog (*Erinaceus europaeus*) is distributed over Ireland, Britain and western mainland Europe. The Algerian hedgehog (*Atelerix algirus*) is present in North Africa and was introduced to Spain and several Mediterranean islands [14]. Moreover, the northern white-breasted hedgehog (*Erinaceus roumanicus*) is distributed throughout Central and Eastern Europe, whilst the southern white-breasted hedgehog (*Erinaceus concolor*) is present in Eastern Europe and Southwestern Asia [15]. The long-eared hedgehog (*Helmiechinus auritus*) maintains part of its predominately Middle Eastern range in Cyprus and Ukraine [13].

Although hedgehog density has been reported to be up to 35% lower near roads [16], road-killed hedgehogs are a very familiar sight across Europe and are frequently the main mammal roadkill recorded in citizen science projects and expert multispecies roadkill surveys [17,18]. For example, an estimated 113,000–340,000 *E. europaeus* individuals are killed on roads every year in the UK [19] and The Netherlands [20], and 230,000–350,000 individuals every year in Belgium [21]. Comparisons between short-term studies are difficult as roadkill rates can fluctuate with changes in hedgehog density, road conditions and traffic volume [22]. Alternatively, long-term roadkill data are valuable to observe changes in temporal behaviour or monitor population trends [23]. For example, Recihholf [24] and Müller [25] found hedgehog road mortality to have steadily decreased since the 1970s in Germany, and Wilson and Wembridge [26] found similar patterns in the UK since 2001. It is claimed that these changes reflect the marked declines over the past two decades of *E. europaeus* in several countries across Europe [25,27]. *A. algirus* has also shown reduced abundance and local extinctions in its introduced range in Europe [14]. However, sufficient hedgehog population data to identify declines are currently limited to the UK [28,29], The Netherlands [30] and, to a lesser extent, Denmark [31] and Germany [25]. Traffic-related mortality has been implicated as a significant component of hedgehog population declines and also constitutes a welfare concern [8,19,24,32]. In recent times, the field has used nationwide monitoring schemes such as “Project Splatter”, a citizen science study in the UK that collates such data [33]. Studies using nationwide data have demonstrated broad spatiotemporal patterns; hedgehog roadkill hotspots are associated with suburban areas and grassland, as well as the breeding season in late spring and early summer [22,32]. Records of hedgehog road mortality have also been used to estimate annual road mortality [19,20,21], track epizootics [22] and have the potential to estimate population abundance [34,35]. Substantial gaps in knowledge remain, however, about whether roads affect long-term population persistence. Likewise, the use of appropriate techniques to evaluate the complexity of the impact (e.g., population modelling using collected demographic data) have received little attention [36].

Investigating the population-level impacts of road mortality is of both theoretical and applied importance. It is likely that Europe is already the most fragmented continent due to transport infrastructure [2,37] and road networks continue to expand rapidly. In the UK alone, an average of 70,000 km of new roads are built every year [38] and many existing roads are modernised or widened throughout Europe [39]. Road development, however, is not consistent across European countries [37]. Coupled with the assertion that road mortality is the leading cause of human-induced vertebrate mortality on land [3], road ecology is a critical frontier of applied scientific research. As several European hedgehog species are declining and disproportionately represented in roadkill records [21], understanding how important road mortality is for population trends is a necessary step for hedgehog conservation. This review aims to consolidate the current knowledge on the consequences of road mortality for the viability of hedgehog populations in Europe. We used online databases to search for and appraise published, peer-reviewed articles on hedgehog road ecology, complemented by government reports on road statistics. This review synthesises information on the possible direct role that road mortality plays in population declines. It then discusses the individual-level risk of road mortality and the contribution of hedgehog–vehicle collisions to much-discussed fragmentation effects and associated genetic heterozygosity. Finally, this review identifies opportunities for road mitigation for hedgehogs, current knowledge gaps and priorities for future research.

## 2. Does Road Mortality Really Reflect Population Persistence?

It is difficult to confirm or refute the impact of road mortality on population trends because survival probabilities depend on a complex set of inter-related factors [40]. Several criteria exist to evaluate the ecological effects of road mortality. For example, the total number of road-killed animals must be considered in the context of population size [19], reproductive output, immigration and emigration rates [41] and whether WVCs are compensatory or additive to other forms of mortality [5]. To date, research has only partially met these criteria. Recent year-round studies have evidenced an average 0.001–3.65 hedgehog casualties/km/year for all European species across several European countries (Table 1). 

Counts of the hedgehogs killed on roads indicate the extent of (lethal) collisions with vehicles and can be used to quantify differences between species, countries and road types if the survey methodology is clearly described. However, they do not indicate the relative importance of traffic-related deaths in the context of populations as a whole [5], and there are issues of standardisation between studies due to differences in study design, effort, frequency and duration. Notably, these issues include accurately accounting for variable carcass persistence [51,52]. Examining the proportion of a population killed on roads every year is more informative. Previous studies of *E. europaeus* have calculated that traffic casualties amount to 9–26% of the total (nationwide) population size in The Netherlands [53] and 10–30% in the UK [19], assuming the population estimates are accurate [28]. At the local scale, previous studies have used capture–mark–recapture methods to identify an annual loss of 3–22% of local *E. europaeus* populations on roads in Sweden [54] and 24% in Poland [46]. Examining the proportional loss at the local scale is instrumental for targeted conservation action. This is because the impact of roads may be different between local populations due to regional variation in habitat type, quality, population densities and road networks [55].

Another promising indication of the population-level effects of road mortality is to compare it with mortality from other sources and identify its contribution to cumulative annual mortality. This can be used to assess the impact of traffic collisions on the mortality–recruitment ratio [5]. *E. europaeus* is the most studied hedgehog species worldwide and mortality of the species has been investigated using radio-tracking methods [43], capture–mark–recapture methods [54] and data from rescue centres [56] (Table 2). It should be noted that the small sample sizes in the reported studies in Table 2 and their study design can skew the relative importance of a cause of death, and that there will naturally be local variation in the occurrence of each mortality factor. Although the studies are an important first step in refining an understanding on mortality, the results should be interpreted with caution. The studies suggest that road traffic is consistently in the top three most common causes of death for hedgehogs, alongside illness and natural predation, supporting the narrative that traffic mortality potentially places substantial pressure on population dynamics. The magnitude of this effect will depend on the ability of populations to compensate for additional mortality by increased survival and/or reproduction, for example, with second litters [57]. Determining how plausible compensation is for hedgehogs is hampered by a lack of data on female hedgehog fecundity, such as the proportion of females that breed successfully, the mean number of litters per female annually and mean litter size, as well as juvenile survival rates. However, the evidence for ongoing hedgehog declines suggests that compensation might not be occurring [27]. It is likely that the declines of hedgehog populations across Europe are a result of a combination of factors. For example, intensified agricultural practices, molluscicide and rodenticide poisoning, badger predation and loss of habitat have also been raised as important correlates of reduced population density and local extinction risk [24,28,58,59,60]. Disentangling the relative impact of factors to population demography, which is likely to be area-specific, remains a principal goal to improve hedgehog conservation. 

## 3. The Risk of Road Mortality Is Not Equal. Which Are the Risk-Prone Individuals?

The risk of road mortality over time varies spatially and between individuals in a population [67]. Differential risk is a function of risk per crossing, which largely depends on animal crossing speed, traffic volume and road width, multiplied by the frequency of crossing. This is associated with individual responses to roads and biological characteristics, such as reproductive strategy and pre-hibernation foraging [68]. Individual-based movement patterns cause different exposure to traffic in the environment [41], which has important repercussions for reproductive output [10]. For example, for species such as hedgehogs that have a promiscuous mating system and maternal natal care, adult females have a more important role in population growth than males [69]. Moreover, the frequency distribution of age-at-death in a population is central to life history evolution and population dynamics [10].

No studies have empirically examined the individual-based risk of road mortality over time for hedgehogs, nor the potential variation in carcass detectability or persistence between different age groups. Current knowledge relies on limited data on the sex ratio and age structure of casualties. During a study of *E. europaeus* over 259.5 km of road in Ireland, Haigh et al. [42] revealed that 65% (67 out of 103) of those killed on roads were male. Moreover, Haigh et al. [70] tested several techniques to age hedgehogs, such as dentary bone analysis, jaw and hind foot length. These produced accurate age assessments and identified that the mean age of road-killed hedgehogs was 1.94 years. These findings were similar to those of Goransson et al. [71], who found that 80% of *E. europaeus* traffic casualties in Sweden were males who had survived one winter. To understand the significance of sex- and age-specific road mortality to population dynamics, these figures should be considered in the context of the number of individuals in that sex/age class in the wider population. Moreover, it is possible that, due to their small size, juvenile hedgehogs are readily scavenged or not detected during driving surveys.

The majority of hedgehogs are reproductively active in their second year (after one successful hibernation) [54,70]. Although research into the road mortality of different sexes and age groups is sparse, the majority of studies indicate that reproductively active males are most commonly killed on roads. Male hedgehogs have larger home ranges and nightly movements than females [14,72], particularly during the breeding season [73]. This would, all other conditions being equal, increase the number of roads that males must cross each night. Conversely, females are most likely to be involved in traffic collisions in autumn after intensive natal care as their net-movement increases to build fat reserves for hibernation [42]. The removal of reproductively active individuals carries a greater threat to hedgehog population viability because it can skew the age ratio and cause a decline in recruitment [74]. On the one hand, the disproportionate loss of adult males may not be as consequential for population growth as adult female deaths [5]. On the other hand, males are more commonly killed before or during breeding season, unlike females [42]. There is a possibility that fewer males successfully contribute to the gene pool and the relatedness in a population increases over time. If severe enough, this may cause a decrease in population fitness associated with inbreeding depression [75] (see Section 4), although research on the topic remains limited.

## 4. The Role of Road Mortality in Fragmentation Effects

Habitat fragmentation by transport infrastructure and the associated development has become one of the greatest threats to biodiversity [39]. The consequences of road-induced fragmentation for the integrity of natural environments are well-researched [76,77]. Several different, yet not mutually exclusive, mechanisms restrict animal dispersal across roads—lethal road collisions, the avoidance of the road or roadside habitat and the inability to traverse the road or nearby area, such as due to a central median or parallel drainage ditch [78]. Road mortality is likely to act as a filter to movement for many species, rather than an absolute barrier, as animals may be able to make successful journeys across the road, even across large roads and bridges [79]. For hedgehogs, road mortality is considered a more severe restriction to dispersal on smaller roads. For example, *E. roumanicus* in Bulgaria [33] and *E. concolor* in Turkey [80] were shown to have greater casualty rates on quieter, regional roads than highways. This may result from quieter roads allowing more crossing attempts [58], having fewer physical barriers than major roads and/or their placement in areas with higher hedgehog densities. In severe cases, increased road mortality could lead to death rates exceeding birth rates, which may change a local population to a sink [81].

Road mortality has been shown to be the largest contributor to population fragmentation [81,82], albeit not always [3]. It is possible that physical barriers such as roadside fencing and road avoidance behaviour cause fragmentation via more stringent restrictions to movement. Both barriers and road avoidance behaviour are particularly common on roads with higher traffic volumes and speeds [78]. Dowding et al. [58] reported avoidance of foraging near roads, but not of crossing quieter roads, by *E. europaeus*. Moreover, Rondinini and Doncaster [83] compared observed *E. europaeus* movements in Southampton, UK, with “random walks” and identified clear road avoidance behaviour that increased with road width (and associated higher traffic). In corroboration with Rondinini and Doncaster [83], a traffic volume of 3000 vehicles/day (common for busy urban roads) in New Zealand led to the isolation of *E. europaeus* populations [22].

This combined effect of road mortality and avoidance for fragmentation is readily explained by traffic flow theory, which postulates a positive and asymptotic relationship between traffic volume and roadkill counts. Road mortality will increase with rising traffic volume until reaching an asymptote, when the busy roads (with greater noise levels) form complete barriers and are avoided, or the roads suppress population size and reduce the number of individuals crossing roads [84]. It is clear that roads constitute semi-permeable barriers for hedgehogs and that the extent of fragmentation is context-specific.

### Biomolecular Insights into Fragmentation

Recent advances in genetic approaches have bridged the gap between molecular and road ecology to address the chronic impacts of fragmentation [85]. Insights into the genetic effects of hedgehog population fragmentation have grown since the development of eleven nuclear microsatellite primers (genetic markers) for *E. europaeus* by Becher and Griffiths [86] and Henderson et al. [87]. The markers have been used to genotype several closely related hedgehog species and can identify genetic similarities between individuals and, therefore, the level of inbreeding [15]. The variability of genetic markers is particularly important for small mammals such as hedgehogs, where fragmentation is likely to act at microspatial scales [88]. Braaker et al. [89] reported that two main rivers and major transport infrastructure (a four-lane highway and railroads) separate three genetic clusters of the *E. europaeus* population in Zurich. Moreover, combined movement models and microsatellite data indicated that fragmentation and high resistance in the urban matrix of Zurich, predominately from highways, footpaths, buildings and water bodies, contribute to the genetic structure of the hedgehog population at the local level, i.e., within clusters [89]. The weak correlation between genetic structure and geographical distance in several additional hedgehog studies indicates that linear infrastructure restricts gene flow enough to affect genetic heterozygosity [88,90,91]. However, the hedgehog’s promiscuous mating system and ability for heteropaternal superfecundity (a litter fertilised by different males) may partly counteract the genetic effects of isolation [92]. Inbreeding coefficients would be reduced as a litter can consist of several half-siblings. The reality of this, however, remains untested, and Barthel [93] reported potentially early signs of inbreeding in *E. europaeus* subpopulations in Berlin. A promising, relatively unused strategy for examining population isolation is genetic pedigree analysis, which uses microsatellites to detect migration rates (e.g., across roads) and local geographies of closely related individuals. This forms a quantitative tool to identify the likelihood of inbreeding and whether the population is acting as a sink population [76,94].

## 5. Potential Road Mitigation Measures for Hedgehog Populations

As road construction and traffic volumes continue to grow, accommodating the increase in human activity without jeopardising the viability of wild populations remains a major challenge. Approaches for sustainable infrastructure development should tackle both the local (mortality and habitat degradation) and landscape (fragmentation and population viability) impacts of roads, yet there is no simple solution or decision-making framework [11]. A growing number of legal imperatives, such as Article 10 of the European Union’s Habitat Directive (92/43/EEC) and the National Environmental Policy Act (1969), as well as international guidelines, such as the United Nations’ Sustainable Development Goals, motivate transport planners to safeguard habitat connectivity and ecosystem functioning. This means newer major roads, in particular those built in Central and Eastern Europe, often have integrated wildlife crossings, such as underpasses or overpasses [39]. Minor roads, however, receive less attention despite the majority of road networks consisting of these low-traffic roads [82]. The range of mitigation measures can be classified using four main criteria; road crossing structures, traffic calming measures, habitat management and configuration of the road network [95].

### 5.1. Road Crossing Structures

Exclusionary fencing is a dominant strategy to impede an animal’s attempt to cross a road. However, fencing was shown to cause a 30% reduction in *E. europaeus* population viability in The Netherlands by intensifying population isolation [96]. Instead, combining fencing with road tunnels or green bridges such as overpasses is widely advocated for many species [36,97,98]. This method strives to reduce barrier effects by providing both a reduction in road mortality and conserving or increasing landscape permeability [5]. Several studies have documented varied levels of crossing structure use by *E. concolor* in Greece [99], *E. europaeus in* Spain [44,100], Portugal [101], the UK [102,103] and Poland [104,105], and *Erinaceinae* sp. in Spain [106] (see review by De Vries [107]). This variation of use is likely due, in part, to differences in tunnel design, location and surrounding habitat, suggesting that the uptake of mitigation depends on optimality of species-specific features. For example, hedgehogs have been shown to frequent tunnels with a greater openness ratio (short in length, high and wide) nearer urban areas [101]. Moreover, previous studies demonstrate that hedgehogs avoid areas with predator (badger *Meles meles*) odour, although the avoidance did not always persist [108,109]. Badgers are known to utilise road tunnels [103], sometimes very regularly, and whether this negatively influences hedgehog use of road mitigation structures remains unknown.

### 5.2. Traffic Calming Measures

Crossing structures are often concentrated at clusters of roadkill [110]. However, this hotspot approach is contentious; several authors propose that a lack of road mortality may signal a previously declined population or a population that exhibits high road avoidance behaviour [111,112]. If so, the necessity for mitigation to assist in population recovery or protection is overlooked. Similarly, the fencing associated with crossing structures could block locations of frequent successful crossings if inappropriately placed. Instead, smaller-scale traffic calming measures that increase driver awareness may be equally effective and substantially cheaper. These aim to enhance preferred crossing sites, which do not necessarily correspond with roadkill hotspots, in order to dissuade the use of riskier crossing locations [7,81]. Traffic calming measures adopted in the past include speed bumps, speed restrictions and warning signs [95]. These initiatives may be particularly effective for hedgehogs given that they frequently attempt to cross quieter roads [58]. Whilst a reduction in speed would be expected to result in a substantial reduction in roadkill [113], the realised effect depends on whether drivers adhere to the speed regulations, which can be difficult to govern [114], and whether, even at a slower speed, a driver can see and avoid a small animal at night.

### 5.3. Habitat Management

Additional mitigation possibilities include managing roadside habitats by increasing habitat quality, local connectivity [95] and changing road verge management [115]. These improve the core habitat and allow individuals to locate sufficient resources whilst crossing fewer roads. Several authors recommend removing or reducing shrubbery in central medians to reduce road mortality [116,117] (but see [118]). The use of central medians by fauna has not been well-studied and, if they are in fact beneficial to animal movement across a road, their removal may exacerbate barrier effects [78]. Modifying hedgerows, which act as conduits of hedgehog movements, near roads is also likely to be an important action. For example, Huijser [53] identified that, out of 942 traffic victims, 20–27% and 140% more *E. europaeus* road casualties were found in areas where hedgerows and railroads, respectively, were perpendicular to roads rather than parallel. Therefore, how roads and local landscape features are orientated in relation to one another warrants consideration.

### 5.4. Road Configuration

In Western Europe, many major roads were built more than 40 years ago with little consideration for wildlife [37]. Retrofitting crossing structures can be an expensive undertaking, and their construction is often logistically challenging [119]. It is therefore essential to consider how landscape configuration can be designed to meet the needs of human settlements, associated road systems and habitat networks simultaneously [120]. Previous multispecies simulation studies have reported that road mortality rates and population persistence were improved when traffic volume was concentrated on fewer roads [121,122]. Surprisingly, van Strien and Grêt-Regamey [119] reported opposite results for hedgehogs. These studies reinforce the significance of whole landscape planning; the high rates of new road development in Central and Eastern Europe provide the opportunity to consider road configuration and maintain suitable habitat matrices for *E. roumanicus* and *E. concolor* [17,49].

## 6. Current Knowledge Gaps and Future Directions

Major impediments to furthering knowledge on hedgehog road ecology are the high labour and monetary costs linked to collecting relevant data for at least one population—that is, road casualty rates, movement and population structure data (and optionally genetic information). Moreover, although GPS devices are increasingly utilised for movement studies [10,123], including for hedgehogs [89,93], the high initial costs often reduce sample sizes and lead to results with poor statistical inference (see [124] for full review). Understanding the ramifications of hedgehog road mortality is further hindered by the lack of basic biological and ecological knowledge on some species such as *E. concolor*, as well as uncertain rigor of population and road casualty estimations for other species. Current population estimates are from citizen science surveys and extrapolations of presence-only density estimates in different habitat types [125,126]. The assumptions associated with these methods make estimates of population size equivocal [19]. Improved population estimates are critical to validate existing findings and could be achieved by large-scale collaborations or more standardised citizen science, such as using camera traps and random encounter methods [127,128]. Moreover, roadkill estimates of many species are likely to be underestimated due to scavengers removing carcasses and varying carcass detectability due to factors such as carcass decay, the driver’s speed and the animal’s body size [81,129]. As a result, raw carcass data must be corrected for carcass persistence and detection probability to obtain accurate estimates of the number of animals killed on roads, as demonstrated by Péron et al. [130] and Santos et al. [52]. Similarly, it is likely that a small proportion of hedgehog–vehicle collisions do not result in instantaneous death and that a hedgehog’s delayed traffic-induced death off the road is not counted. The possible role of wildlife hospitals in affecting estimates of mortality rates and genetic fragmentation is also important to consider. Particularly common for *E. europaeus* in Western Europe, wildlife rehabilitators care for and release injured hedgehogs that would otherwise die [64]. While this is undoubtedly valuable for the species’ conservation, future road ecology analyses must consider confounding factors such as these.

Of particular significance is that studies seldom examine road mortality in the context of a population’s intrinsic growth rate. Considering growth rates reveals less of a “snapshot” of mortality and determines whether populations can sustain current and future road casualty rates. Future research should explicitly model the sensitivity of population growth curves to sex- and age-specific road mortality, using methods such as population viability models and elasticity analysis [41]. Population modelling could be further used on existing data sets, such as from nationwide citizen science projects, to accurately estimate yearly road mortality or, for populations with both road mortality and density estimates, an estimate of local demographic compensation. Another informed approach could incorporate population density, the sex and age of casualties and other sources of mortality into the framework of compensation-additive mortality [131]. This explores whether road mortality is compensatory and removes the already “doomed surplus” in a population or is additive by increasing total mortality [55]. For example, if road-killed individuals have a poor body condition (e.g., they are affected by parasites or other diseases), the severity of road mortality is reduced as their likelihood of long-term survival is low regardless of traffic [43].

The efficacy of road mitigation measures for wildlife is rarely tested; this poses significant constraints on justifying mitigation efforts and adapting strategies for maximum benefit. Many studies are either too short or adopt study designs that cannot demonstrate causality to population viability, such as gene flow or lasting reductions in road mortality [132]. In the future, studies should employ long-term monitoring of mitigation measures and before-and-after-control-impact (BACI) or control-impact experimental designs, where possible. These studies allow for changes in the investigated population parameters, such as density, sex ratio or genetic diversity, to be soundly attributed to the mitigation measures [133]. Future research should also present more holistic mitigation recommendations by examining socioeconomic factors such as vehicle and pedestrian travel efficiency [119] and the cost-effectiveness of strategies [98] (Table 3). The challenge of accommodating both hedgehog and anthropogenic demands on the landscape highlights the crucial role of interdisciplinary and collective thinking in road ecology [11].

## 7. Conclusions

As hedgehogs remain a prominent victim of WVCs and road infrastructure continues to expand in Europe [38,39], evaluating whether hedgehog populations are vulnerable to the long-term negative impacts of roads is urgently needed. The literature presents several evaluative criteria for this purpose, including proportional loss, differential risk between demographic groups and the fecundity of the remaining population. Previous studies are in general agreement that adult males are more prone to road mortality and that hedgehog–vehicle collisions can disrupt population dynamics, for example, by fragmentation. However, barriers exist to understanding whether this translates to population decline and to disentangling the relative impact of road mortality on population viability compared to other factors. These difficulties remain the primary challenges for hedgehog conservation throughout Europe. Future research should prioritise the inclusion of sex- and age-specific fecundity and survival rates in population models and analyses. This review highlights the importance of long-term monitoring and robust experimental design such as BACI for effective decision-making by conservation practitioners and policy makers. Moreover, considerations of wildlife must be integrated into the early planning stages of road construction to meet the goals of sustainable development. Collaboration between ecologists, engineers and spatial planners is not only good practice, but likely to be indispensable in achieving a reduction in the conflict for space that characterises the 21st century.

## Figures and Tables

**Table 1:** Estimates of the mean number of hedgehogs killed on roads per kilometre per year in different European countries. Data are derived from systematic, year-round studies.

Species	Country	Year(s) of Study	Mean Number of Road Casualties/km/Year	Surveyed Road Types	Habitats along Survey Route	Reference
*E. europaeus*	Ireland	2008–2010	0.001	National and regional roads	Residential, rural, woodland and built-up areas	[42]
	Finland	2004–2005	0.007	National, regional and local roads	Residential, forests and built-up areas	[43]
	Spain	2001–2003	0.76–1.42	National roads	Forests	[44]
	Slovakia	2000–2002	1.6	National, regional and local roads	Agricultural land, forests and nature preserves	[45]
*E. europaeus* and *E. concolor*	Poland	2001–2003	0.07	National, regional and local roads	Arable land and built-up areas	[46]
*A. algirus*	Lanzarote	2010–2011	0.65	National, regional and local roads	Urban areas and Badlands	[18]
*E. roumanicus*	Bulgaria	2017	0.06–0.08	National and regional roads	Rural areas	[47]
	Bulgaria	2015–2017	0.23	National roads	Arable land, riparian vegetation and forests	[17]
	Ukraine	2000–2009	0.73	National, regional and local roads	Urban areas and forests	[48]
	Slovakia	2008–2012	1.52–2.68	National roads	Agricultural land, grasslands, forest, riparian and built-up areas	[49]
	Ukraine	2002–2005	3.65	National roads	Marshes, forests and urban areas	[50]

**Table 2:** Percentage of deaths by different sources for studied *E. europaeus* individuals in Europe.

Location	Year of Study	Number of Individuals	Roads	Natural Predation (Badger and Fox)	Unnatural Predation (Dog and Cat)	Illness ^a^	Poison	Other ^b^	Unknown	Reference
UK and The Netherlands	1999	~83580	8.8	Not included	2.3	58.5	4.3	26.0	Not included	[56]
UK	1988	109	78.0	Not included	Not included	Not included	2.8	1.8	17.4	[61]
UK	1981	22	18.2	31.8	13.6	Not included	4.5	31.8	Not included	[62]
UK	1992	8	25.0	75.0	Not included	Not included	Not included	Not included	Not included	[63]
UK	2019	7	43.0	43.0	Not included	14.0	Not included	Not included	Not included	[64]
UK	1998	7	57.0	14.0	Not included	14.0	Not included	14.0	Not included	[65]
Finland	2016	106	72.6	Not included	0.9	20.7	Not included	1.8	4.0	[43]
Denmark	2019	9	Not included	Not included	22.0	22.0	11.0 ^c^	44.0	Not included	[66]

^a^ includes parasitological, pathological (e.g., starvation and gangrenous limb) and bacteriological findings (e.g., *Salmonella*); ^b^ includes drowning, injury from agricultural or garden tools and fire; ^c^ speculated but not confirmed.

**Table 3:** Summary of published findings, as well as gaps in the literature and recommendations for future research as discussed in this review.

Published Findings	Gaps in Understanding as Revealed by This Review	Directions for Future Research as Recommended by This Review
Traffic collisions may cause an annual loss of 3–24% of a local hedgehog population, and 9–30% of a nationwide population [19,46,53,54]. Road mortality is consistently in the top three contributors to total mortality [40,60,61].	The accuracy of current local and total population estimates. Whether populations can compensate for road mortality with increased survival and/or fecundity.	Establishing standardised surveys for improved population estimates. Long-term population studies to evaluate road mortality in the context of population growth.
Hedgehog roadkill is disproportionately clustered in suburban areas and consists predominately of males and adults [28,30,39,68].	Whether carcass detectability and persistence vary between age groups. How road and habitat characteristics influence road mortality risks between demographic groups over time.	Studies into the road crossing behaviour of different demographic groups. Evaluating the consequences of sex- and age-specific road mortality on hedgehog population trends.
Hedgehog populations appear particularly vulnerable to fragmentation effects [30,81]. Hedgehog populations exhibit distinct genetic substructure, often in relation to linear infrastructure [86,90,91].	Whether the hedgehog’s promiscuity and heteropaternal superfecundity can lessen the impacts of isolation on genetic structure.	Establishing isolation effects from roads, such as using inbreeding coefficients or genetic pedigree analysis.
Exclusionary fences alone are not an appropriate mitigation measure for hedgehog road mortality [96]. Hedgehogs infrequently use crossing structures [96,98,101].	The population-level responses to mitigation measures. Whether the use of crossing structures by badgers impacts their efficacy for hedgehogs. Whether traffic-calming methods are an effective and cheaper option for road mitigation.	Quantification of population viability in relation to mitigation using BACI or control/impact studies, such as using roadkill counts, population density and gene flow. Integration of ecological and socioeconomic perspectives on road mitigation and construction.
